# Building a nomogram plot based on the nanopore targeted sequencing for predicting urinary tract pathogens and differentiating from colonizing bacteria

**DOI:** 10.3389/fcimb.2023.1142426

**Published:** 2023-05-17

**Authors:** Shengming Jiang, Yangyan Wei, Hu Ke, Chao Song, Wenbiao Liao, Lingchao Meng, Chang Sun, Jiawei Zhou, Chuan Wang, Xiaozhe Su, Caitao Dong, Yunhe Xiong, Sixing Yang

**Affiliations:** ^1^ Department of Urology, Renmin Hospital of Wuhan University, Wuhan, China; ^2^ Department of Cardiovascular Surgery, The Affiliated Hospital of Qingdao University, Qingdao, China

**Keywords:** nomogram, nanopore sequencing, LASSO regression, urinary tract infections, asymptomatic infections

## Abstract

**Background:**

The identification of uropathogens (UPBs) and urinary tract colonizing bacteria (UCB) conduces to guide the antimicrobial therapy to reduce resistant bacterial strains and study urinary microbiota. This study established a nomogram based on the nanopore-targeted sequencing (NTS) and other infectious risk factors to distinguish UPB from UCB.

**Methods:**

Basic information, medical history, and multiple urine test results were continuously collected and analyzed by least absolute shrinkage and selection operator (LASSO) regression, and multivariate logistic regression was used to determine the independent predictors and construct nomogram. Receiver operating characteristics, area under the curve, decision curve analysis, and calibration curves were used to evaluate the performance of the nomogram.

**Results:**

In this study, the UPB detected by NTS accounted for 74.1% (401/541) of all urinary tract microorganisms. The distribution of ln(reads) between UPB and UCB groups showed significant difference (OR = 1.39; 95% CI, 1.246–1.551, p < 0.001); the reads number in NTS reports could be used for the preliminary determination of UPB (AUC=0.668) with corresponding cutoff values being 7.042. Regression analysis was performed to determine independent predictors and construct a nomogram, with variables ranked by importance as ln(reads) and the number of microbial species in the urinary tract of NTS, urine culture, age, urological neoplasms, nitrite, and glycosuria. The calibration curve showed an agreement between the predicted and observed probabilities of the nomogram. The decision curve analysis represented that the nomogram would benefit clinical interventions. The performance of nomogram with ln(reads) (AUC = 0.767; 95% CI, 0.726–0.807) was significantly better (Z = 2.304, p-value = 0.021) than that without ln(reads) (AUC = 0.727; 95% CI, 0.681–0.772). The rate of UPB identification of nomogram was significantly higher than that of ln(reads) only (χ^2 = ^7.36, p-value = 0.009).

**Conclusions:**

NTS is conducive to distinguish uropathogens from colonizing bacteria, and the nomogram based on NTS and multiple independent predictors has better prediction performance of uropathogens.

## 1 Introduction

Urinary tract infection (UTI) is among the most frequent infectious diseases in the community and healthcare setting and usually classified into asymptomatic bacteriuria (ABU), uncomplicated UTI, and complicated UTI (cUTI) to assess the infectious severity and decide whether to apply antimicrobial drugs actively (Wagenlehner et al., 2020). ABU in adults without risk factors corresponds to commensal colonization, and active treatment is not recommended in the latest European Association of Urology (EAU) guidelines (Bonkat, 2022). Similarly, pathogenic detection is not strongly recommended for uncomplicated UTI patients unless empirical antimicrobial therapy is ineffective (Hooton et al., 2021). Despite that the obvious benefits of antibiotic use to cUTI patients has been testified, the more sobering reality is that overuse and misuse have led to a growing problem of drug resistance in uropathogens (Eliakim-Raz et al., 2019; Quan et al., 2021). As can be seen, recent guidelines and consensus have distinguished between urinary tract pathogenic bacteria (UPB) and colonizing bacteria (UCB), as their handling attitudes are markedly different.

Urine dipstick and microscopic analysis are often used for qualitative preliminary screening, after which etiological detection is still required. Urine culture is of great significance for the pathogenic diagnosis and effective treatment of UTI (Bonkat, 2022). However, culture methods are not always sensitive and accurate but always with a huge time cost (McDonald et al., 2017; Dauwalder et al., 2021). Statistically, the sensitivity of culture is approximately 30%, and negative results cannot completely rule out UTI; even multiple tests are not uncommon. Moreover, further studies showed that the small counts of *Escherichia coli* in midstream urine were too low to form sufficient colony unit (CFU) for diagnosis in urine culture and were still highly predictive of acute cystitis. In contrast, the enterococci and group B streptococci frequently cultured from midstream urine were rarely isolated from urine sampled from the bladder through catheterization (Gupta et al., 2017). Therefore, urine culture cannot effectively differentiate UPB from UCB. Clinicians make a diagnosis, heavily relying on a combined analysis of clinical and medical history and test manifestations. Previous studies have constructed nomograms, a simple and accurate visualization tool, based on multi-factors for predicting UTI, but there are problems of poor performance and cumbersome variables. Li et al. used culture results strictly as a diagnostic criterion for UTI; the UTI patients was only 13.5% (171/1271), and negative results of urine culture could not completely exclude UTI (Li et al., 2023). Yang et al. established a preoperative nomogram to predict postoperative urosepsis for negative preoperative urine culture patients. Unfortunately, there is no postoperative etiological detection to verify urosepsis (Yang et al., 2023). It is not recommended to predict UPB completely out of touch with etiological detection.

Recent genomic sequencing techniques allow the highly sensitive detection of every microorganism in any specimen, and their expanded application in urine testing has changed the traditional dogma that urine of healthy individuals is sterile (Gu et al., 2021). The latest Nanopore Targeted Sequencing (NTS) offers a powerful option to overcome the above clinical diagnostic challenges, which has been reported for the rapid, accurate, and comprehensive detection of respiratory viruses and endophthalmitis pathogens (Rusk, 2019; Wang et al., 2020; Huang et al., 2021). There is an article documenting the first clinical attempt of NTS to detect multi-system pathogens, where the mentioned NTS detected 20 common UPB, but the urine sample size was extremely limited (n=74), much less the interpretation of the sequencing results for determining the pathogenicity of detected bacteria (Fu et al., 2022). Therefore, this study is the first to analyze NTS data of urinary tract microorganisms in detail. To ensure that the NTS data reflected uropathogens as accurately as possible, we innovatively combined NTS results with clinical information related to uropathogenic infections to screen out independent predictors for the identification of UPB or UCB and constructed a visualized nomogram to calculate the likelihood of infection with uropathogens.

## 2 Materials and methods

### 2.1 Study population

The study was a retrospective analysis consisting of consecutive patients in the Department of Urology, Renmin Hospital of Wuhan University from June 2020 to August 2021. The inclusion criteria were patients whose urine had been detected by NTS, urine culture and routine tests before antibacterial therapy at admission, and NTS indicated that there may be urinary microorganism(s) or even uropathogen(s), no matter one kind or more. Potential factors associated with UTI including possible baseline characteristics, medical history, and laboratory test data were collected for regression analysis.

The exclusion criteria were as follows: (1) anti-infective treatment before admission or sample collection; (2) clinical data obtained from non-midstream urine and cystostomy or nephrostomy status; (3) lack of urine routine or culture on admission; (4) patients whose urine collections for NTS, urine culture, and urine routine tests were performed at different time periods; (5) the urine culture suggested a contaminated sample or was completely inconsistent with NTS; and (6) patients with incomplete clinical data.

All personal information was masked during the process of analysis and publication. This retrospective study has obtained the informed consent from the Ethics Committee of the Renmin Hospital of Wuhan University (WDRY2022-KS006), which abandoned the requested written informed consent.

### 2.2 Etiological detection methods and routine variables

#### 2.2.1 NTS technology

After specimen collection, the work was carried out according to the following steps. Professional operators extracted samples’ DNA using Sanure DNA extraction kit (Sansure Biotech, China). Second, library preparation through polymerase chain reaction (PCR) process target amplified bacterial 16s rRNA and fungal internally transcribed spacer 1/2 (ITS1/2), and then, 1D ligation kit SQK-LSK109 (Oxford Nanopore, UK) was used to mixed amplification products in the ratio of 10:3. Libraries were sequenced using MinION platform (Oxford Nanopore, UK). Finally, software including Guppy software (version 6.0.0), Porechop software (version 0.2.4), and Blast software (version 2.9.0) were used to process sequencing data and map to NCBI FTP 16S rDNA/ITS reference database. Through sequencing and data processing, a urine sample generated abundant sequencing reads.

Reads number represented the number of sequencing reads with >90% identity matched to each target region of the genome in database used to describe corresponding bacteria or fungi. To facilitate the calculation and display of data differences, the number of reads is converted to its logarithmic form, abbreviated as ln(reads).

The number of urinary tract microbiological species (NUP) was determined by the classification of sequencing reads if there were multiple matches referring to bioinformatic analysis above.

#### 2.2.2 Urine culture

The pathogenic bacteria were cultured and isolated according to the standard procedure, and the isolated UPB were identified and tested for drug sensitivity by applying the automatic microbiological analyzer Phoenix-100 (Becton, Dickinson and Company, USA) and auxiliary identification card or drug sensitivity card. Bacteriuria was defined as the presence of at least 10^5^ colony-forming units per milliliter. Results were categorized as positive and negative.

#### 2.2.3 Routine variables

Potential predictive variables were chosen based upon review of the literature and guidelines, including the fasting plasma glucose (FPG) and routine urine test at admission (Eliakim-Raz et al., 2019; Bonkat, 2022). FPG, urine pH, urine white blood cells (WBCs), epithelial cells count, urine casts, and pathological casts were continuous numeric variables. The color, clarity, urine glucose, ketonuria, protein, occult blood, nitrite, and leukocyte enzymes of urine were collected by categories.

### 2.3 Diagnosis and definitions

The clinical diagnosis of UTI is referred to clinical guidelines and published literature and based on a combination of clinical features and results of laboratory testing (Wagenlehner et al., 2020; Bonkat, 2022).

#### 2.3.1 Identification of UPB

Primary microorganism in NTS was considered as UPB in patients who was required to have at least one sign or symptom of UTI, lower urinary tract symptoms (LUTS) involving frequency or urgency or dysuria, flank pain including costovertebral angle tenderness, fever of 37.3°C or worse, with any one of the following positive urine laboratory test results, nitrites, leukocyte esterase, protein quantification >2 g/24 h, centrifugal urine sediment WBC count more than five cells/HP, and midstream urine culture showing bacterial growth over 10^5^ CFU/ml.

#### 2.3.2 Diagnosis of UCB

The patient with the single presence of potential symptoms above or a positive urine test result alone was considered to have ABU or non-UTI symptoms, whose primary microorganisms in NTS are defined as UCB.

### 2.4 Statistical analysis

The continuous numeric variables that coincided with normal distribution were expressed as mean ± standard deviation (X ± SD). Categorical variables were described as count and percentage. Univariate logistic regression and McNemar–Bowker test were used for intergroup statistical analysis.

All study indexes and variables were introduced into the least absolute shrinkage and selection operator (LASSO) regression to screen out potential predictors for the identification of UPB and UCB. Subsequently, multivariate logistic regression was used to determine the independent predictors and build a model using stepwise backward regression analysis, which visualized with a nomogram. The model was internally validated by resampling K-fold cross-validation (K = 5, times = 400) and calculating the resampled AUC.

The receiver operating characteristic curve (ROC), area under the ROC curve (AUC), and the decision curve analysis (DCA) were used to graphically assess the discriminating performance and clinical utility of the nomogram. Plotting calibration curves were used to assess the relationship between the actual and predicted probabilities of UPB infections. Each predictor’s contribution in the nomogram was measured by the partial chi-square statistic minus the predictor degrees of freedom and visually shown in a bar chart. DeLong’s test was used for comparison of two correlated ROC curves. Data were analyzed by SPSS software (version 26.0) and R software (version 4.1.2), and two-tailed p<0.05 was considered statistically significant.

## 3 Results

### 3.1 Patient characteristics

In the study, 541 patients at admission were consecutively enrolled for analysis. According to diagnostic criteria and detecting results, the primary microorganisms detected by NTS in 401 (74.1%) patients were UPB, and 140 (25.9%) people were UCB. Baseline characteristics of two groups patients are presented in **Table 1**.

**Table 1:** The baseline characteristics of included patients with UTI at admission.

Characteristics	Overall	UCB (140)	UPB (401)	OR	CI	p
Age, means (SD)	58.3 (14.3)	55.2 (16.5)	59.4 (13.2)	1.02	1.007–1.035	0.003
Man, n (%)	339 (62.7)	96 (68.6)	243 (60.6)	0.7	0.468–1.061	0.094
Hypertension, n (%)	147 (27.2)	34 (24.3)	113 (28.2)	1.22	0.785–1.906	0.373
Diabetes, n (%)	74 (13.7)	14 (10.0)	60 (15.0)	1.58	0.855–2.934	0.144
LUTS, n (%)	115 (21.3)	17 (12.1)	98 (24.4)	2.34	1.342–4.08	0.003
Hematuria, n (%)	63 (11.6)	18 (12.9)	45 (11.2)	0.86	0.478–1.536	0.604
Flank pain, n (%)	209 (38.6)	63 (45.0)	146 (36.4)	0.7	0.474–1.034	0.073
Fever (>37.3°C), n (%)	21 (3.9)	2 (1.4)	19 (4.7)	3.43	0.789–14.923	0.1
Urinary calculi, n (%)	358 (66.2)	100 (71.4)	258 (64.3)	0.72	0.474–1.098	0.128
BPH, n (%)	125 (23.1)	30 (21.4)	95 (23.7)	1.14	0.715–1.812	0.585
Urological neoplasms, n (%)	85 (15.7)	25 (17.9)	60 (15.0)	0.81	0.485–1.351	0.418
UAFA, n (%)	87 (16.1)	24 (17.1)	63 (15.7)	0.9	0.538–1.508	0.691
Urological endoscopic surgery, n (%)	160 (29.6)	35 (25.0)	125 (31.2)	1.36	0.878–2.103	0.169
Nephrostomy or cystostomy, n (%)	14 (2.6)	2 (1.4)	12 (3.0)	2.13	0.47–9.631	0.327
NUP, means (SD)	1.9 (1.1)	2.2 (1.2)	1.9 (1.1)	0.77	0.651–0.906	0.002
Ln(reads), means (SD)	7.6 (1.9)	6.8 (1.8)	7.9 (1.8)	1.39	1.246–1.551	0
Positive urine culture, n (%)	167 (30.9)	16 (11.4)	151 (37.7)	4.68	2.678–8.182	0
FPG, means (SD)	5.3 (1.7)	5.2 (1.6)	5.4 (1.7)	1.1	0.954–1.276	0.183
Gross hematuria, n (%)	36 (6.7)	10 (7.1)	26 (6.5)	0.9	0.423–1.92	0.788
Cloudy urine, n (%)	446 (82.4)	121 (86.4)	325 (81.0)	0.67	0.39–1.157	0.152
Glycosuria, n (%)	46 (8.5)	5 (3.6)	41 (10.2)	3.07	1.19–7.945	0.02
Ketonuria, n (%)	39 (7.2)	8 (5.7)	31 (7.7)	1.38	0.62–3.084	0.429
PH, means (SD)	6.2 (0.7)	6.2 (0.5)	6.3 (0.8)	1.21	0.922–1.586	0.171
Proteinuria, n (%)	281 (51.9)	60 (42.9)	221 (55.1)	1.64	1.11–2.414	0.013
Occult blood, n (%)	420 (77.6)	100 (71.4)	320 (79.8)	1.58	1.017–2.454	0.042
Nitrite, n (%)	85 (15.7)	8 (5.7)	77 (19.2)	3.92	1.842–8.348	0
Leukocyte enzymes, n (%)	391 (72.3)	94 (67.1)	297 (74.1)	1.4	0.921–2.122	0.116
WBC count, means (SD)	699.8 (2315.7)	324.1 (1481.8)	830.9 (2531.7)	1	1–1	0.043
Epithelial cells count, means (SD)	8.8 (15.8)	8.3 (17.5)	9.0 (15.2)	1	0.99–1.016	0.641
Urine casts, means (SD)	0.4 (1.0)	0.3 (0.6)	0.5 (1.1)	1.29	0.958–1.724	0.094
Pathological casts, means (SD)	0.2 (0.6)	0.1 (0.2)	0.2 (0.7)	1.52	0.773–3.001	0.223

LUTS, lower urinary tract symptoms, BPH, benign prostatic hyperplasia; UAFA, urological anatomical and functional abnormalities; NUP, number of urinary tract microbiological species; FPG, fasting plasma glucose; WBC, urine white blood cells

In the basic information, the mean age of the enrolled patients was 58.3 ± 14.3 years, and 339 patients (62.7%) were men. In terms of chronic underlying diseases and specific medical history, 147 (27.2%) people suffered from hypertension and 74 (13.7%) from diabetes, 358 (66.2%) patients had urinary tract calculi, 125 (23.1%) patients had benign prostatic hyperplasia (BPH), 85 (15.7%) had urologic neoplasms, 87 (16.1%) had urological anatomical and functional abnormalities (UAFA), 160 (29.6%) had a history of urological endoscopic surgery, and 14 (2.6%) patients had nephrostomy or cystostomy. Some patients had existing more than one urogenital disease or chronic comorbidities. Flank pain was recorded in 209 (38.6%) patients, LUTS in 115 (21.3%), hematuria in 63 (11.7%), and fever (>37.3°C) in 21 (3.9%).

### 3.2 NTS detection results

**Figure 1:**
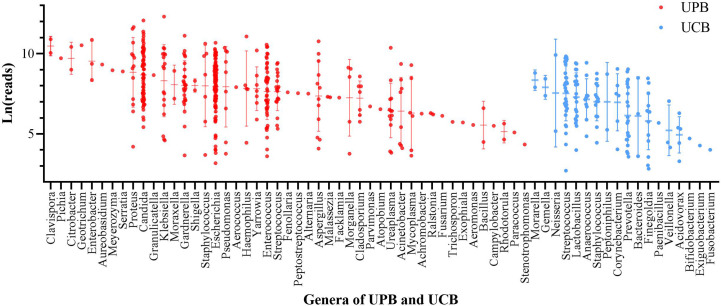
The ln(reads) distribution of primary microorganism of 541 enrolled patient. Microorganisms were divided into urinary tract pathogens (UPB) and colonizing bacteria (UCB) groups, classified by genus, and ranked according to the mean value of ln(reads). Each dot represented one sample, red for UPB and blue for UCB.

In univariate logistic regression, ln(reads) and NUP were significantly different between the UPB and UCB groups. To further explore the NTS test results and urinary tract microorganisms, we showed ln(reads) distribution of primary microorganisms classified by genus in 541 urine samples. According to above criteria, microorganisms were divided into UPB and UCB groups, classified by genus, and ranked according to the mean value of ln(reads) (**Figure 1**). At the genus level, in order of detection rate, *Escherichia* spp., *Candida* spp., *Enterococcus* spp., *Gardnerella vaginalis*, *Streptococcus pneumoniae*, *Klebsiella* spp., *Proteus* spp., *Ureaplasma* spp., *Pseudomonas* spp., *Acinetobacter* spp., and *Staphylococcus aureus* were the most common UPB groups found in NTS. Consistent with previous studies, *E. coli* was still the highest detection rate pathogen at the level of species. UCB groups did not belong to the UPB group as mentioned above and were not deemed to cause symptoms of infections, including non-uropathogenic *Streptococcus* spp., e.g. *Streptococcus mitis*, *Lactobacillus* spp., *Prevotella* spp., non-uropathogenic *Staphylococcus* spp., e.g., *Staphylococcus epidermidis*, *Finegoldia magna*, and *Anaerococcus* spp.

**Figure 2:**
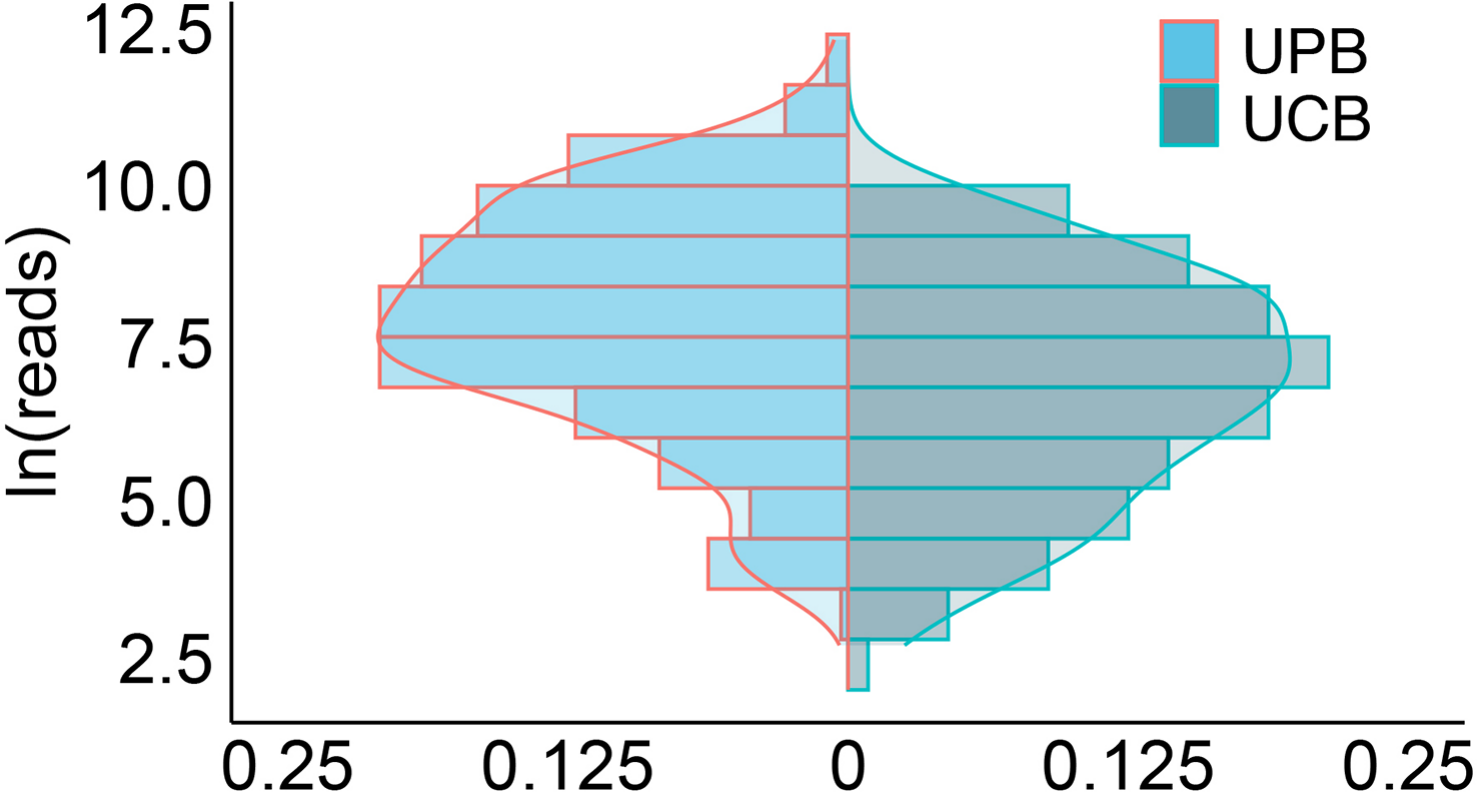
The histogram symmetrically displayed the distribution of ln(reads) in the UPB and UCB groups. The horizontal coordinate represented the percentage, the vertical coordinate represented ln(reads), the red line represented uropathogens, the blue line represented colonizers, and the two sets of ln(reads) data were symmetrically distributed on both sides of X=0.

**Figure 3:**
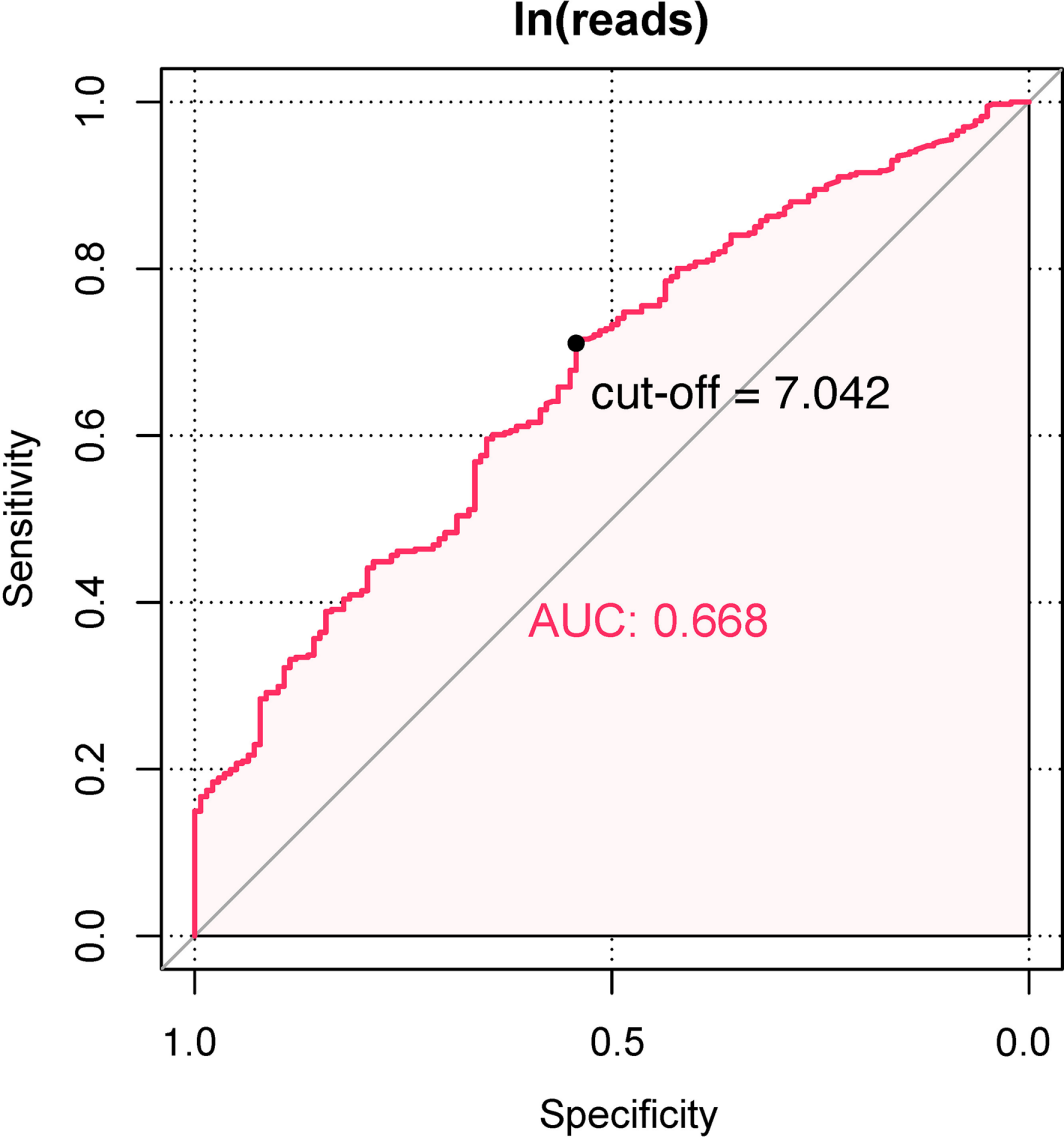
The ROC and AUC of ln(reads) to distinguish UPB or UCB.

There was a significant difference in ln(reads) between the UPB and UCB groups graphically displayed with a symmetrical histogram (**Figure 2**), which possessed a preliminary ability to identify UPB and UCB (AUC=0.668), and the corresponding cutoff value was 7.042 (**Figure 3**). AUC value was not entirely satisfactory, and we tried to find independent predictors to identify UPB from UCB to improve diagnostic efficiency.

### 3.3 Predictor selection

**Figure 4:**
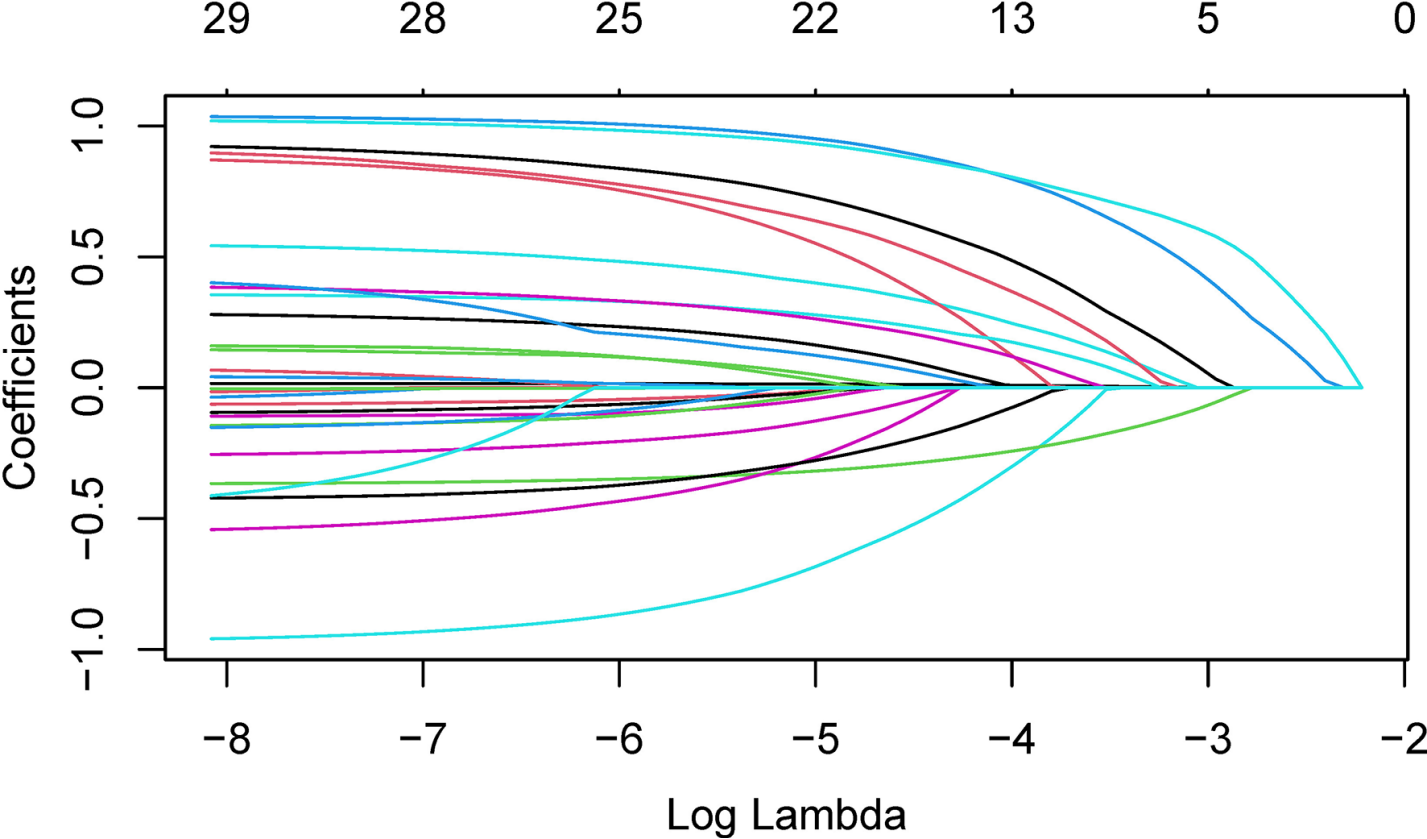
Plots for coefficient distribution of 33 variables in LASSO regression. The horizontal coordinate indicates the logarithm of the penalty parameter λ, the vertical coordinate represents the coefficient of potential predictor, and colored curves represents the coefficients changing trajectories of the predictors as the penalty parameter λ increasing.

**Figure 5:**
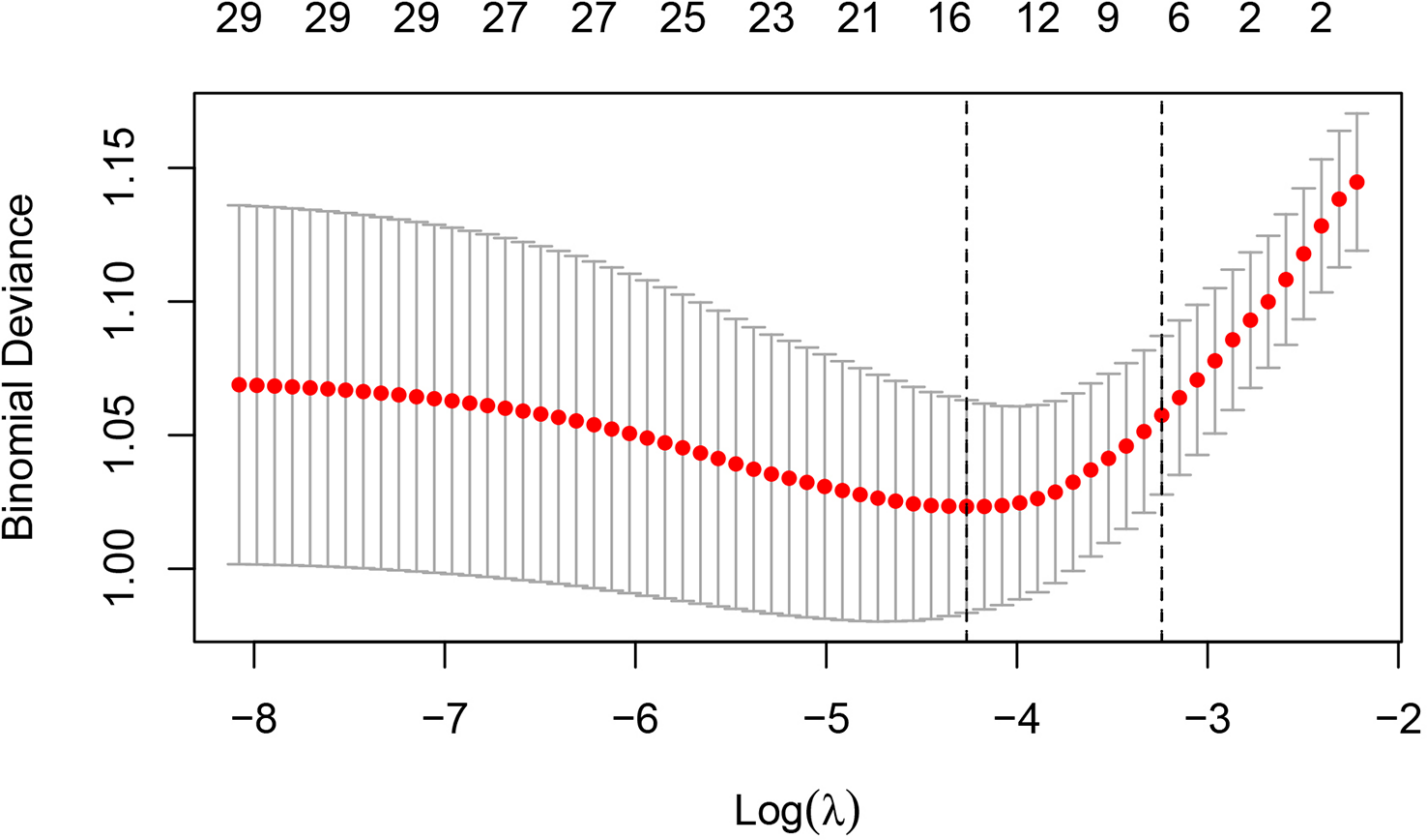
Cross-validation results for the penalty parameter in LASSO regression. The left dashed line corresponded to the value of the parameter log(λ) and the number of variables with the minimum variable loss error. When log(λ) = −4.26, 14 variables were filtered out.

**Figure 6:**
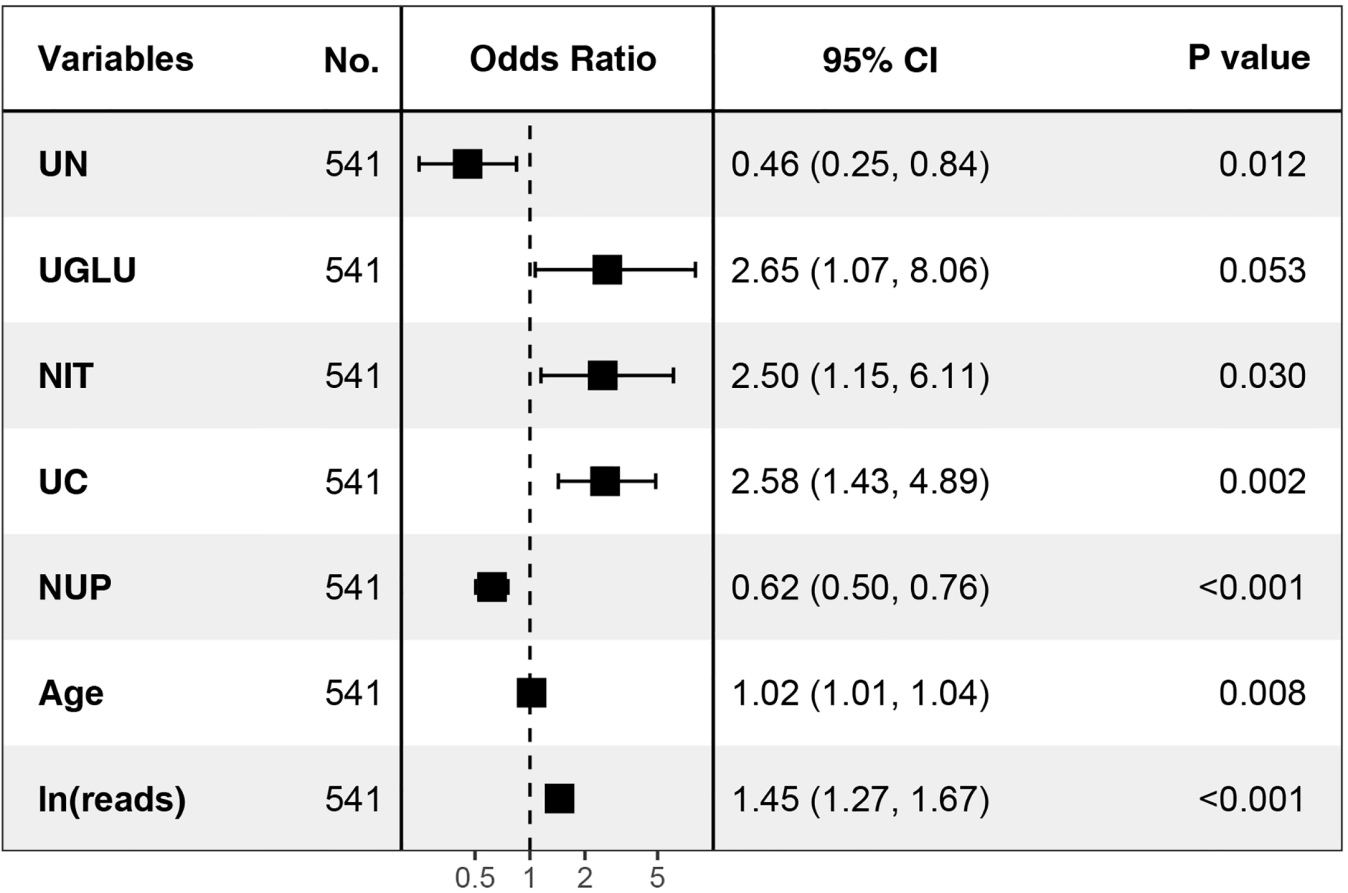
Seven independent predictors in multi-factor logistic regression. UN, urological neoplasms; UGLU, urine glucose; NIT, urine nitrite; UC, urine culture; NUP, number of urinary tract microbiological species.

In order to determine, to the extent possible, the clinical indicators that have an association with the predicted outcome, we performed regression analysis with all the above-mentioned indicators that may be associated with UPB infections. First, 33 UTI-related features were subjected to LASSO regression (**Figures 4**, **5**). UPB infections may be associated with 14 variables: age, LUTS, fever (>37.3°C), urological neoplasms, urological endoscopic surgery, ln(reads), NUP, urine culture, urine clarity, urine glucose, urine protein, urine occult blood, nitrites, and urine casts. These 14 variables were included in a multifactorial logistic regression model, yielding seven variables as independent predictors for distinguishing UPB from UCB, including age, urological neoplasms, NUP in NTS testing, ln(reads), urine culture, urine glucose, and nitrites. Their OR values and 95% confidence interval (CI) are shown in **Figure 6**.

### 3.4 Nomogram construction and verification

**Figure 7:**
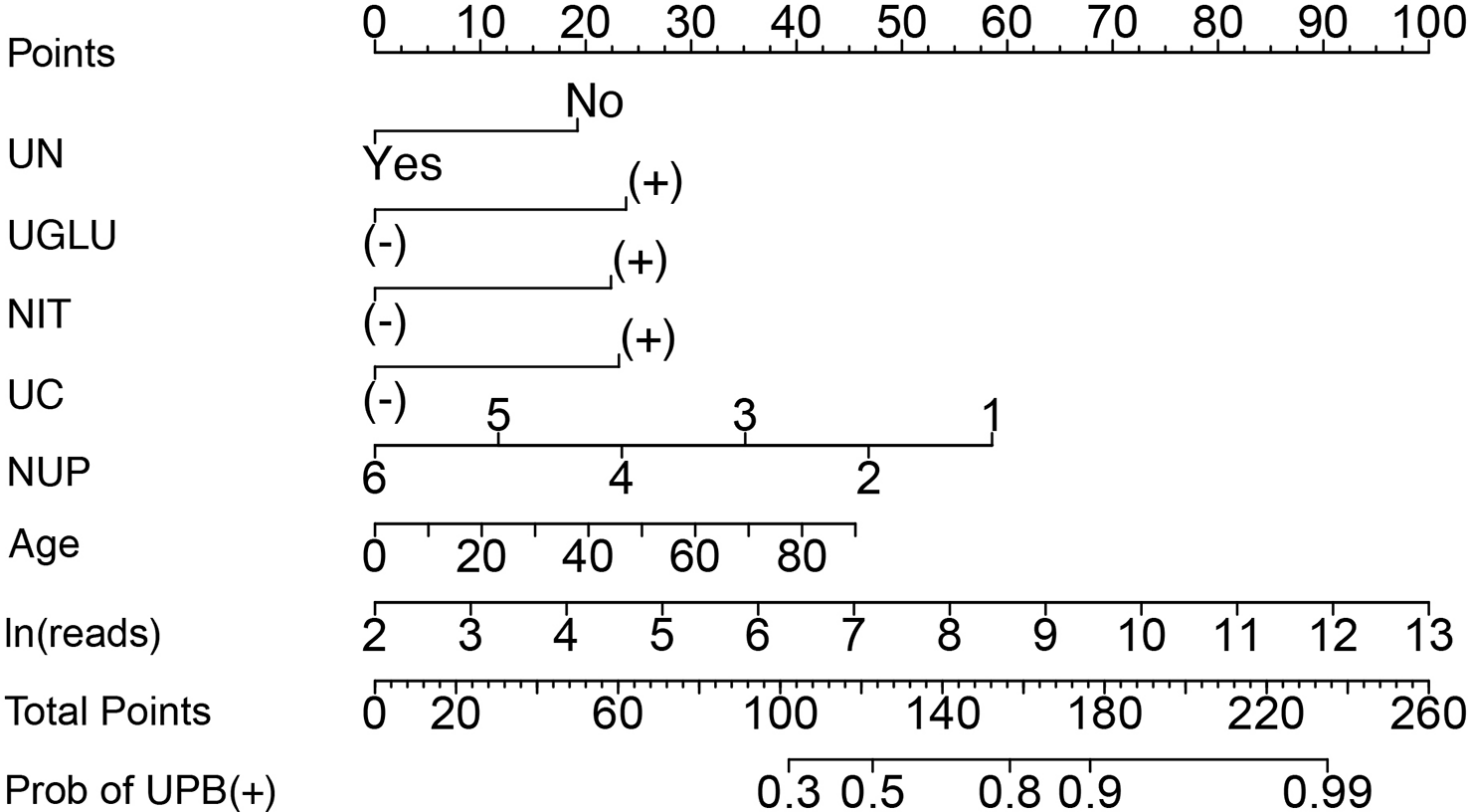
Nomogram to distinguish between UPB and UCB of patients suspected with UTI by calculating the sum of the corresponding scores for each indicator. UN, urological neoplasms; UGLU, urine glucose; NIT, urine nitrite; UC, urine culture; NUP, number of urinary tract microbiological species. ‘+’ or ‘−’ represents positive or negative result for corresponding laboratory test indicator.

A nomogram was constructed with the seven independent predictors to distinguish between UPB and UCB in admitted patients. The nomogram assigned scores to each of the seven variables, and the risk score was calculated based on the coefficients of the logistic model and the values of the expressions corresponding to the seven candidates in the model; the probability of UPB infections for a given patient was computed by summing the allocations for each variable (**Figure 7**).

**Figure 8:**
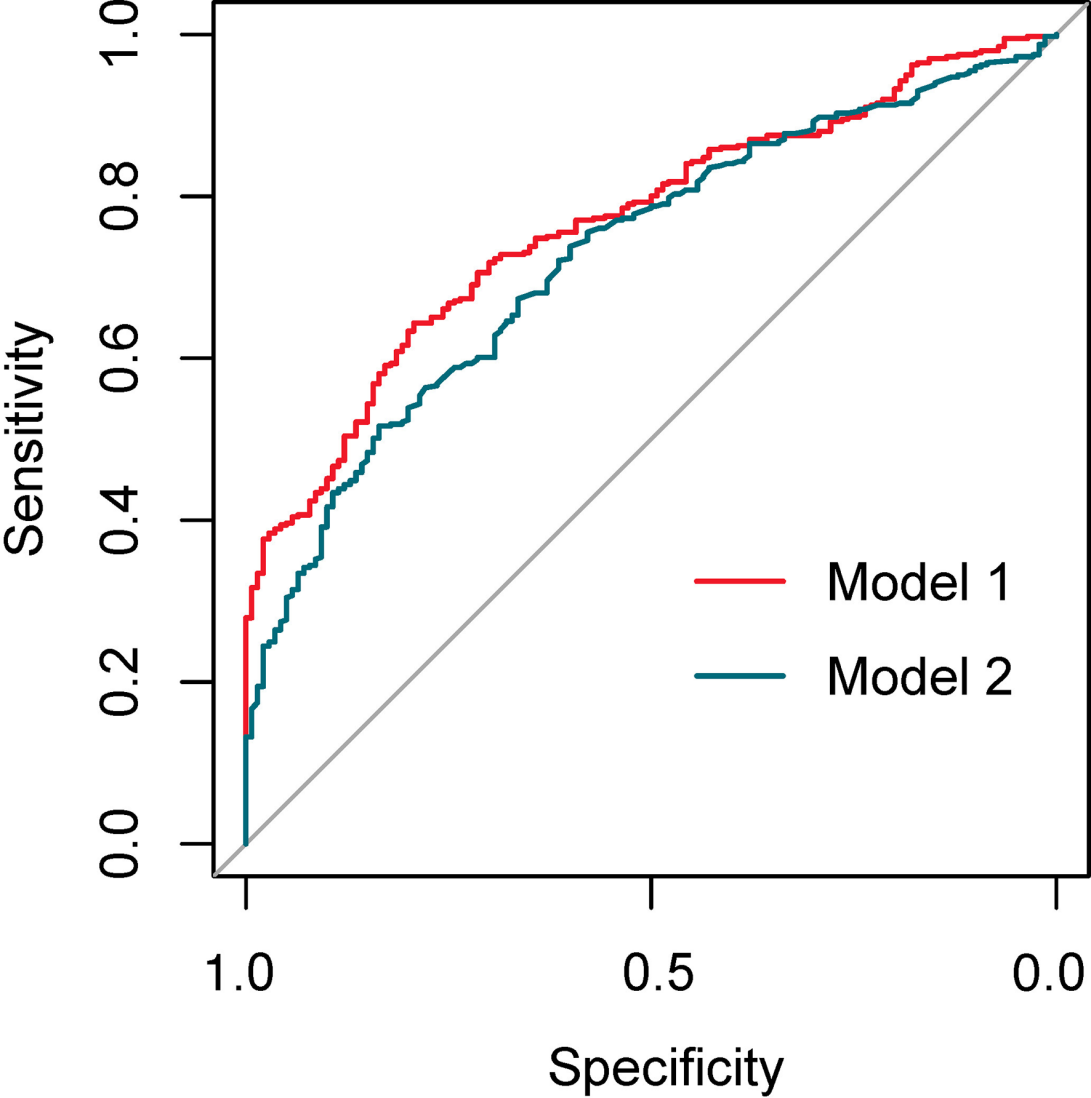
The ROC curves of the models with ln(reads) (Model 1) and without ln(reads) (Model 2).

**Figure 9:**
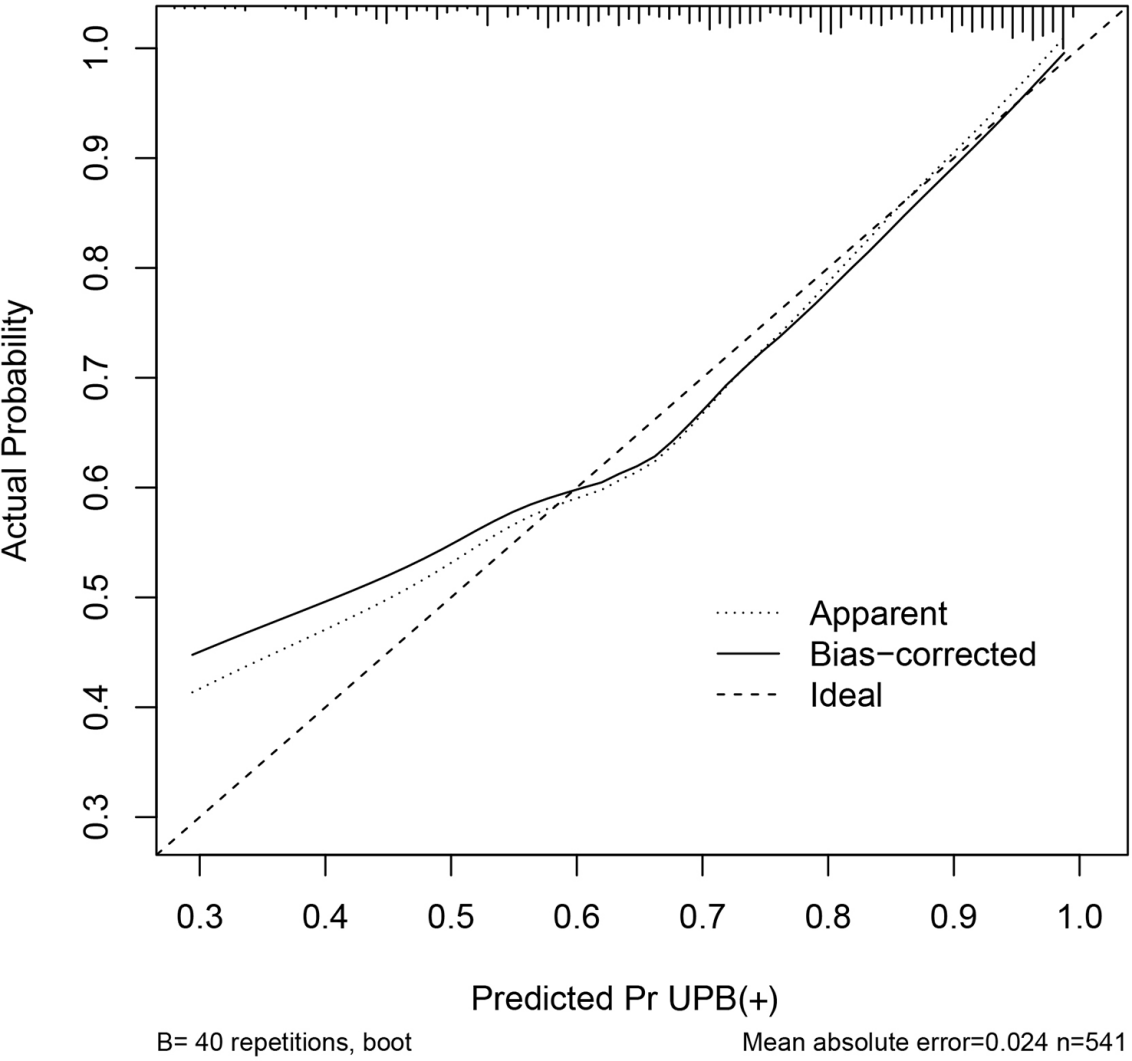
Calibration curve of the nomogram. The horizontal and vertical coordinates represent the predicted probability of uropathogens infection by the nomogram and actual probability of infection, the diagonal line is a reference line representing the predicted probability equaled to the actual probability, and the black line indicates the degree of matching between the predicted and actual results of this nomogram.

**Figure 10:**
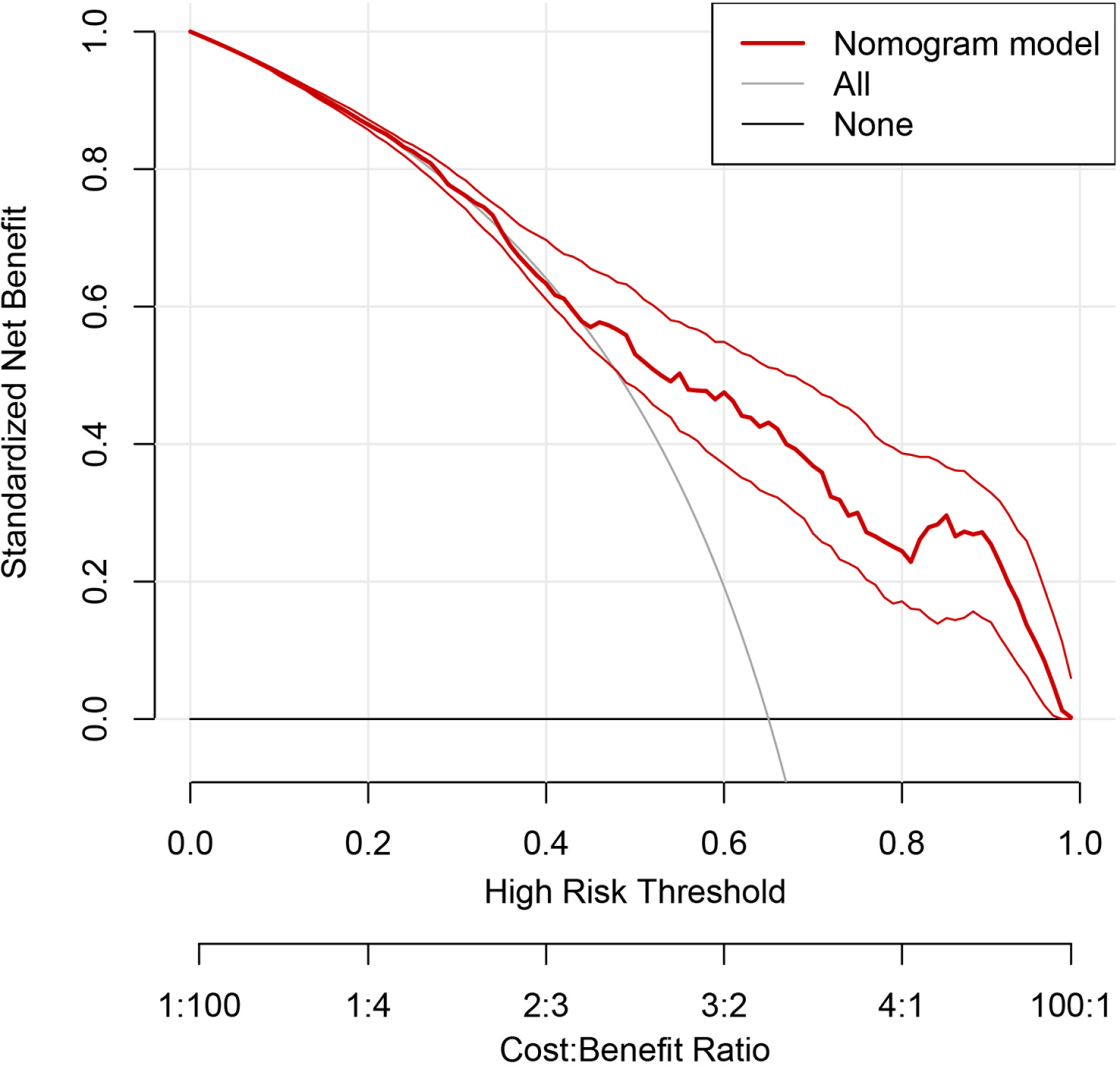
Decision curve analysis (DCA) of the nomogram. The horizontal coordinate represents the threshold probability, above which the predicted outcome was a true positive; the clinical intervention at this point would benefit, there are drawbacks conversely, and the vertical coordinate represented the net benefit after the benefit minus the drawback. The gray curve is the net benefit of clinical intervention for all enrolled patients, the black line at Y=0 represents no clinical intervention for any outcome, so the net benefit is always 0. The thick red line represents the benefit of clinical interventions with reference to the predicted results of the nomogram.

The discriminating degree and performance of the prediction model was evaluated by the ROC curve test (AUC = 0.767; 95% CI, 0.726–0.807; **Figure 8**, Model 1), so did internal validation and the resampling K-fold cross-validation (K = 5, times = 400) of the model (AUC = 0.750). The calibration curve of the model (**Figure 9**) showed an agreement between the predicted and observed probabilities of the nomogram without undesirable deviations. The DCA (**Figure 10**) representing the net benefit of clinical interventions relying on nomogram were higher than that of full or no intervention without the help of nomogram and indicated that the use of this nomogram to identify UPB infections to intervene may provide more benefit than the original treatment strategy. According to the analysis, there would be higher risk of infections with UPB for patients whose total score of all indicators is ≥148, and in turn, <148 means higher likelihood of microbial colonizing in urinary tract. The validation results demonstrated that the nomogram score could be used as a predictor of UPB infections and clinical intervention accordingly.

### 3.5 The role of ln(reads) in nomogram

**Figure 11:**
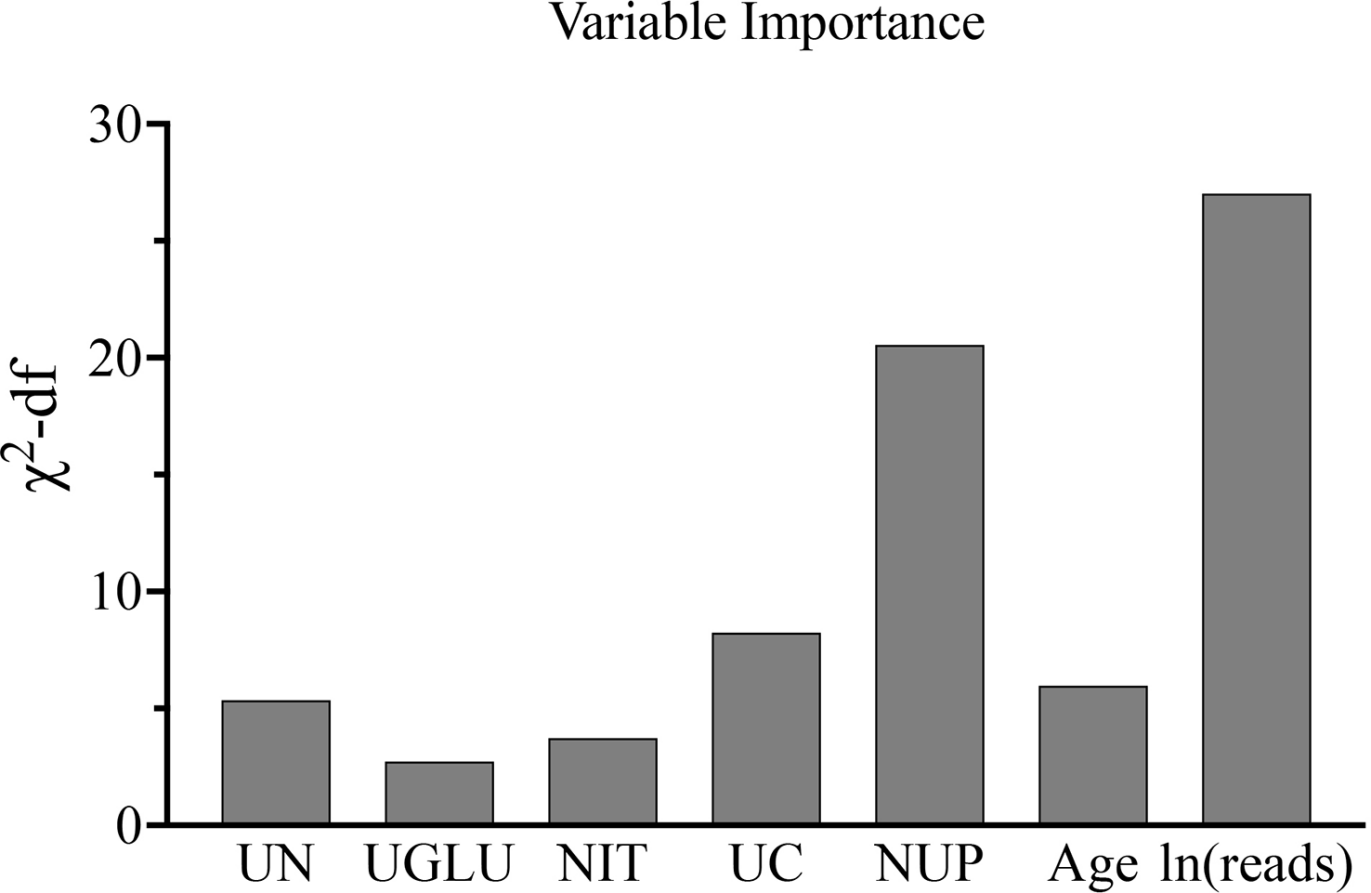
The bar chart showed importance ranking of seven independent predictors of the nomogram.

**Figure 12:**
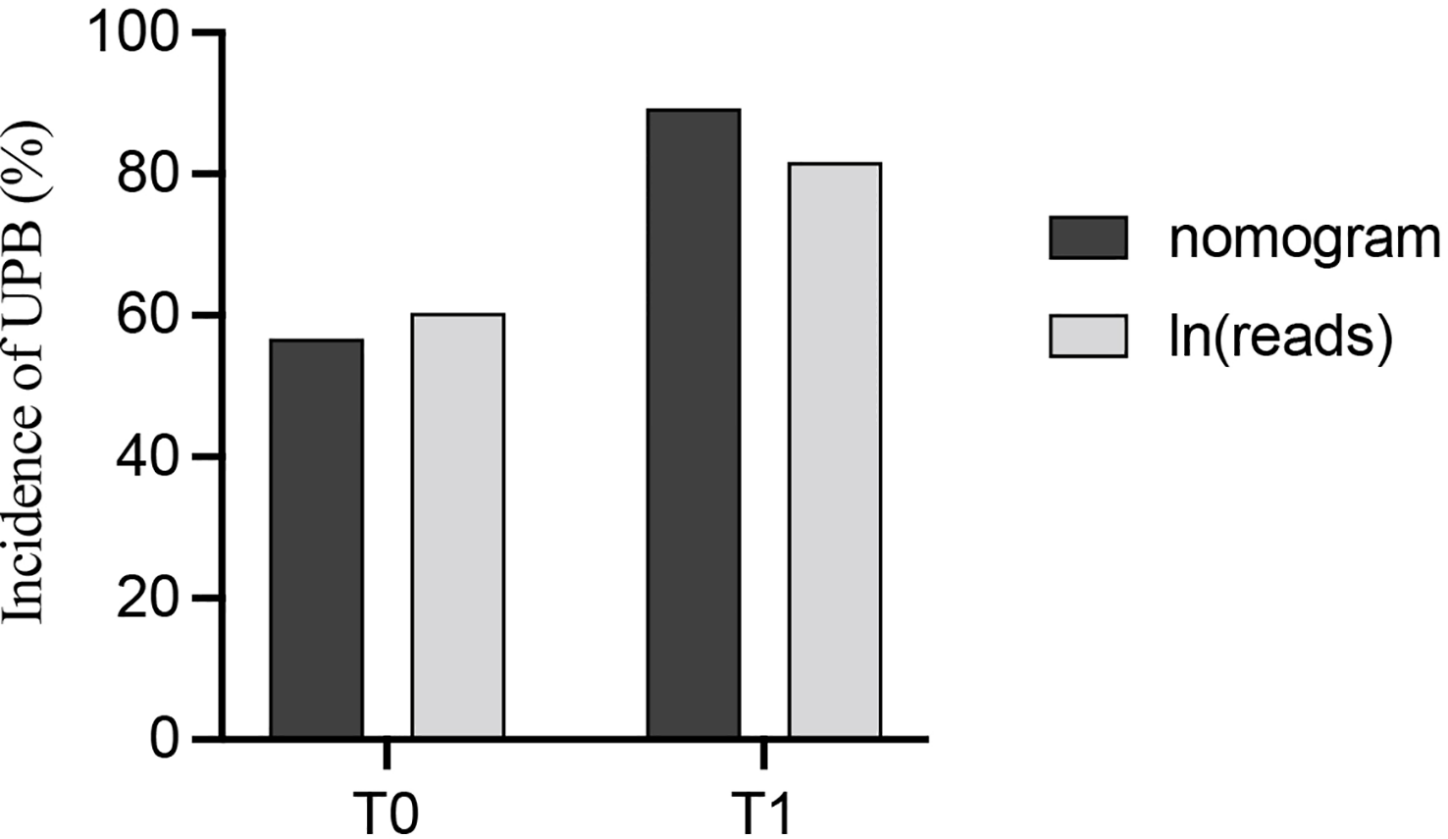
The probability of identifying UPB was compared between the nomogram and ln(reads) alone after grouping with the cutoff value of ln(reads).

Building the bar chart to identify the importance of seven predictor variables found ln(reads) came out on top (**Figure 11**). Following the above methods, we tried to remove the ln(reads) in the model and only used the rest six variables, namely, age, UN, NUP, urine culture, urine glucose, and nitrites, to repeat the modeling process again constructing the simple model (**Figure 8**, Model 2) and compared their predictive performance. The prediction efficiency of nomogram with ln(reads) (AUC = 0.767, 95% CI 0.726–0.807) was significantly better (Z = 2.304, p-value = 0.021) than that without ln(reads) (AUC = 0.727; 95% CI, 0.681–0.772). The ln(reads) of NTS could significantly improve the predictive effect of nomogram. Dividing into two groups according to the cutoff value, it can be found that the nomogram based on NTS can cover more UPB than ln(reads) only (**Figure 12**). The nomogram incorporating multiple factors can further expand the scope of identification based on ln(reads) only (χ^2 = ^7.36, p-value = 0.009).

## 4 Discussion

Nowadays, the popularization of sequencing technology has proved that there are still microorganisms in the bladder urine of healthy individuals; even the concepts of urinary microflora and urinary microbiome have been proposed (Markowski et al., 2019; Yacouba et al., 2022). More than one reference stresses the importance of distinguishing between pathogens and colonizers in the urinary tract environment (Yoshimura et al., 2021; Zhao et al., 2021). Timely and accurate detection of pathogens will contribute to targeted treatment of UTI patients, which can significantly reduce the occurrence of multi-drug-resistant uropathogens and life-threatening urosepsis (Quan et al., 2021). This study was the first to use nanopore targeted sequencing (NTS) for urinary microbiota detection in a large clinical sample size and made the first attempt at interpretation in detail of sequencing results for distinguishing UPB from UCB. The results suggested that NTS could be used to identify UPB to some extent and that a nomogram constructed by combining NTS with other infection-independent predictors could achieve better performance in prediction.

Sequencing technologies are better suited to this job than urine culture (Petersen et al., 2019; Marshall et al., 2021). Some studies have pointed out that reads of metagenomic next generation sequencing (mNGS) can diagnose lower respiratory tract infections and identify the true-positive pathogenic bacteria; however, there is still a hot topic of discussion about efficient clinical applications or precision of mNGS for its short read length (Gasiorek et al., 2019; Liu et al., 2022). Compared with the previous sequencing technologies, nanopore sequencing achieves high throughput and long read length (≥150 kbp), with the advantages of large-scale multiplex sequencing and real-time analysis and can realize timely and comprehensive visualization overall urinary tract microbes (Noakes et al., 2019; Moss et al., 2020). In addition, targeted amplification during library preparation can reduce human host interference and enhance the genomic abundance of microorganisms (Kovaka et al., 2021). To some extent, the advantages of NTS with long read length to sensitively, comprehensively, and accurately detect multi-system microorganisms have been demonstrated (Charalampous et al., 2019; Burgess, 2020; Gilpatrick et al., 2020). Although the value of reads in the identification of UPB and UCB was not extremely satisfactory, the ln(reads) and NUP of NTS occupied the top 2 positions in the variable importance ranking, which effectively validated NTS as a determinant of model’s validity. Hence, the preliminary determination of UPB from NTS results has become possible from a clinical perspective, especially when culture results are not available; the clinical value of the nomogram is more easily demonstrated.

Multi-species of microorganisms in the urinary tract environment were found to be a protective factor in our study; the greater the variety of urinary tract microorganisms, the less likely to be infected with UPB. Horwitz proved that colonization with *E. coli* did not impact bacterial bladder diversity, but subjects who developed infections were all associated with overgrowth of a urinary pathogen and had less diverse bladder microbiota (Horwitz et al., 2015). Florian suggested that while polymicrobial colonization was considered a pre-infection state from a pathophysiological perspective, however, compared to the healthy, the increased risk of UTI was usually due to reduced clearance of UPB or increased UCB (Wagenlehner et al., 2020). Therefore, maintaining a stable UCB group for people who cannot avoid the growth of UCB will help reduce the incidence of UPB infections. As described in the EAU guidelines, urinary growth of bacteria in an asymptomatic individual may protect against superinfecting symptomatic UTI (Neugent et al., 2020; Bonkat, 2022).

We collected UTI-related clinical data for regression analysis and screened out seven independent predictors to construct the nomogram, which showed favorable performance in UPB identification. The two items described above indicates that NTS is the major contributor to this nomogram, and beyond that, urine culture, age, urological neoplasms, urine glucose, and nitrites are additional independent predictors of UPB infections.

In this study, the role of urine culture was to diagnose typical UPB with a pathogen detection rate of 37.7% (151/401); negative results still have the possibility of infection. There is no doubt that urine culture is still an independent factor in determining the pathogenic organism but not the most important with positive results scoring approximately 20 in the nomogram, even negligible in certain cases, while NTS score was apparently higher and dominant. Multi-study results consistent with clinical experience showed that culture methods always seem to lag, and the positive rate would be influenced by potential applications of empirical antibiotics therapy (Wang et al., 2022). Gupta suggested that the role of urine culture is to retrospectively confirm and emphasized the definition of “contaminants” and the threshold of bacteriuria required for the diagnosis needed to be re-discussed (Gupta et al., 2017).

Age was an important risk predictor for the infections with UPB; the likelihood of infections with UPB increased by 1.6% for each additional year of age in patients. Similarly, a study demonstrated that older age was an independent predictor of treatment failure for cUTI patients and risk factors for 30-day mortality (Eliakim-Raz et al., 2019). It might be associated with miscellaneous underlying diseases, dysregulated immune status of host, and the variability in antibiotic resistance pattern (Frisbie et al., 2021). Hence, differentiating UPB from urinary tract microbiota in elderly patients will help to avoid the overuse of antibiotics and reduce the generation of multidrug-resistant strains (Bonkat, 2022).

Regression analysis results found that UCB were more likely to be detected in the urine of patients with urological neoplasms. It is perhaps related to adjuvant intravesical chemotherapy instillations for re-hospitalized bladder urothelial carcinoma patients (Bajic et al., 2019). This result is consistent with Peng’s finding that bacterial richness was increased in genitourinary cancer groups classified at a higher risk of recurrence or progression, indicating that microbial composition may help predict the prognosis of cancer patients (Wu et al., 2018). Notably, available evidence suggests an association between the differences in genitourinary microbial composition or diversity and the development or progression of genitourinary malignancies, with both pathogenic factors and cofactors among microbiota members. Unfortunately, the number of relevant articles has been limited, and the types of differential flora found in different studies vary widely (Yacouba et al., 2022). The more readily accepted view is that the chronic inflammatory response after the early acute UPB infection may be a carcinogenic factor, making the search for preventive factors in UCB group particularly necessary, similar to the preventive role of *Lactobacillus vaginalis* in controlling UPB colonization in women and reducing UTI (Markowski et al., 2019). What is clear is that sequencing technology and statistical analysis will help to study the relationship between the urinary tract microbiota and genitourinary neoplasms, and forthcoming clinical application of NTS will contribute to the subsequent research on this subject.

It has been adequately demonstrated that urine nitrite was beneficial in predicting the infections of UPB, most of which were Gram-negative *Bacillus* in Florian’s statistics concerning the prevalence of uropathogenic bacterial species in cUTI, converting nitrates to nitrites (Wagenlehner et al., 2020). Multiple studies have reported that urine nitrite is associated with UTIs; even preoperative positive urine nitrite is an independent risk factor for postoperative fever after ureteroscopy (Velasco et al., 2020; Ma et al., 2021).

It should be noted that it was glycosuria rather than blood glucose that could predict the infections of UPB in this study; the latter was not significantly different in the two data sets, so did diabetes. Diabetes has been shown to increase the risk of all classification of UTIs, from ASB to cUTI (Gupta et al., 2017). However, glycosuria is believed to provide an optimal environmental condition for bacterial growth (Mohanty et al., 2022). Research showed that glycosuria exposure augmented group B *Streptococcus* epithelial adherence and hemolysis and antimicrobial peptide resistance (John et al., 2021). Islam found that glycosuria rapidly increased gene expression encoding biofilm formation and central metabolic virulence of uropathogenic *E. coli (* Islam et al., 2022). Whether urine glucose plays a greater role in identifying uropathogenic bacteria from colonizers needs more data to be supported.

### 4.1 Limitation

First, it should not be overlooked that NTS is currently unable to perform drug-resistant strain prediction, but the potential exists to identify drug-resistant genes (Arango-Argoty et al., 2019; Xu et al., 2021). Second, there are differences in colonization of different parts in the urinary tract, which cannot be identified in the midstream urine. Third, there is still the possibility of infection caused by colonizing bacteria, and even non-first bacteria in the microorganisms cause disease. For opportunistic pathogens, there should be two states of colonization and pathogenicity, which have not been discussed in this study. Hence, the conclusions of this study cannot be directly used to guide the prevention of infection of UPB from healthy individuals, and better prediction models need to be further developed.

## 5 Conclusions

We dug deeper into NTS data than ever before and demonstrated that NTS was conducive to distinguish uropathogens from colonizing bacteria. Through LASSO and multivariate logistic regression, we screened out seven independent predictors and established a nomogram to determine whether the patient has uropathogenic infection, which showed good performance in internal verification and better prediction effect than NTS only.

NTS was the primary contributor to the validity of this nomogram and showed great potential in etiological detection. The nomogram might contribute to timely detecting uropathogens, in-depth interpretation for clinical applications of NTS, and the research on urinary tract microorganisms.

## Data availability statement

The datasets presented in this article are not readily available because: The full data belongs to the Renmin hospital of Wuhan University, it is not in a public repository. Data is included in part in **Supplementary Table 1**, and further inquiries can be directed to the corresponding authors.

## Ethics statement

The studies involving human participants were reviewed and approved by The Ethics Committee of the Renmin Hospital of Wuhan University. Written informed consent for participation was not required for this study in accordance with the national legislation and the institutional requirements.

## Author contributions

SJ, YW, YX, and SY contributed to conception and design of the study. HK, CSu, JZ, and LM collected the clinical data. YW, CW, and XS performed the statistical analysis. SJ wrote the first draft of the manuscript. CSo, CD, and WL wrote sections of the manuscript. All authors contributed to the article and approved the submitted version.
